# Evaluation of clinical knowledge of drugs causing addiction and associated social determinants among male pharmacy and nursing students in Riyadh, Saudi Arabia – A Cross-Sectional study

**DOI:** 10.1016/j.pmedr.2024.102606

**Published:** 2024-01-16

**Authors:** Omaimah A. Qadhi, Moadeyah Mohammed Alasmari, Ibrahim Nasser Alsulaihim, Wajid Syed, Mahmood Basil A. Al-Rawi

**Affiliations:** a Department of Medical-Surgical College of Nursing, King Saud University, Riyadh, Saudi Arabia; b Department of Maternity and Child Health, College of Nursing, King Saud University, Riyadh, Saudi Arabia; c Department of Pharmacology and Toxicology, College of Pharmacy, King Saud University, Riyadh 11451, Saudi Arabia; d Department of Clinical Pharmacy, College of Pharmacy, King Saud University, Riyadh 11451, Saudi Arabia; e Department of Optometry, College of Applied Medical Sciences, King Saud University, Riyadh, Saudi Arabia

**Keywords:** Drug addiction, Heroin, Stimulants, Complications, Adverse events

## Abstract

Drug abuse is a rising psychological concept in many countries, and its use among individuals is increasing. Therefore, this study aimed to assess the Knowledge and demographic factors associated with drug abuse among male pharmacy and nursing students at King Saud University, Riyadh, Saudi Arabia. This study used a cross-sectional design targeting male entry-level pharmacy and nursing students in their first and second years of Bachelor of Nursing and Doctor of Pharmacy courses. Of them, 85.3 % of the pharmacy and 75.3 % of nursing students thought that cocaine causes drug addiction, followed by heroin (pharmacy 80.7 %; nursing students 71 %), and morphine (pharmacy 75.2 %; nursing students 59.1 %). In this study, 52 % (n = 105) claimed low awareness, whereas 48 % (n = 97) indicated good understanding regarding drug addictions. Furthermore, the mean knowledge score among pharmacy students was higher (7.073 ± 2.570) in comparison to nursing (5.806 ± 2.494) (*t* = 3.540; *p =* 0. 0001). In addition, the father’s occupation was found to be significantly associated with the mean knowledge score of drug addiction (F = 2.667; *p* = 0.034). According to the findings, 52 % of male students had insufficient knowledge about drugs that cause addiction. Age, course of study, and father's occupation all had a substantial impact on knowledge scores. The knowledge score on the complications of addictive substances was not significantly associated with the characteristics of the students (*p* = 0.05). As a result, we advocate for the introduction of educational initiatives that educate students about the harmful consequences of drug addiction and how to avoid issues.

## 1 Introduction

Drug misuse is the most frequently discussed psychological issue among adolescents and teenagers in both developed and developing countries(Richert et al., 2020, Volk, 2016). As the use of drugs for non-medical or mind-altering purposes has become more prevalent in daily life, it has gotten worse and worsened psychological problems(Mobrad et al., 2020, Pates and MCBRIDE, A. J., LI, S., & RAMADAN, R. , 2002). An earlier study from Saudi Arabia indicated that young people frequently use psychoactive substances(Al-Musa and Al-Montashri, 2016, Dabbagh et al., 2021). Cannabis, amphetamine, and central nervous system stimulants were reportedly the most often discovered narcotics(Al-Musa and Al-Montashri, 2016, Dabbagh et al., 2021). On the other hand, psychotherapeutic pharmaceuticals, antihistamines, stimulants, and sympathomimetics were the most commonly abused prescribed OTC medications among students in Saudi Arabia(Al-Musa and Al-Montashri, 2016, Dabbagh et al., 2021) and other developed countries like the USA (United States of America)(Butler et al., 2021, Jones et al., 2020, Lord et al., 2009, Weyandt et al., 2021).

According to a Substance Abuse and Mental Health Services Administration report in 2018, there were more than 17.4 million individuals aged between 12 and 25 years were illegal drug adductors(Jones et al., 2020). However, according to Schulenberg, et al., 2018, study the habit of drug intake begins early in secondary school and is fully developed in college life college students were more likely hit by a variety of drug addictions(J. Schulenberg et al., 2019). College students (45 %) had the highest rate of drug abuse, followed by students in the 12th grade (39 %), 10th grade (30 %), and 8th grade (13 %). Overall, 43 % of people between the ages of 19 and 28 who abuse drugs do so. The most widely used substances among abusers were alcohol (73.7 %), marijuana (42.5 %), Adderall (14.6 %), 3,4-methyl​enedioxy​methamphetamine (Mdma) (7.2 %), cocaine (7 %), Lysergic acid diethylamide (LSD) (6.8 %), and tranquilizers (3.8 %)(J. Schulenberg et al., 2019).

There were bundles of reports that evidenced that using drugs for nonmedical purposes causes addiction and drug dependence, which is mainly responsible for causing serious epidemiological diseases and disturbing quality of life (Organization, 2010, Siriwatanamethanon et al., 2012). However, the most cited factors for illicit drugs among the students were emotional immaturity, lack of self-esteem, poor interest in education or schooling, and a bad relationship with their parents (McCrystal et al., 2007, Zhuravleva, 2001). A recent report from different college students published in September 2020 revealed that the majority of the students were influenced by their friends to abuse drugs followed by curiosity to use, search for fun, or overcome school-related stress to overcome family-related issues and media influence also it was evidenced that most commonly abused drug was marijuana comparing to other illicit drugs (Arria et al., 2017; J. E. Schulenberg et al., 2021, Welsh et al., 2019). Some other studies reported that students used illicit drugs to boost concentration in the studies improve outcomes of the study or enhance alertness. However, earlier reports also revealed that students used a combination of prescription and illicit stimulants for academic performance enhancement (Hildt, Welsh et al., 2019).

Despite drug dependency, continuous use of prohibited substances can bring potential health hazards and evidence continues to support that serious health consequences may arise with many side effects and even death (Aishwaryalakshmi, Sasikala, Sreelalitha, Vigneshwaran, & Padmanabha, 2012). All this is often preventable through offering adequate awareness and knowledge about drug abuse and the lifelong harms associated with it. A gap in knowledge about addiction can cause drug dependency. Distributing the proper information can not only guide society through such events but can also increase drug abuse preparedness which might prevent them from using it in the future. In addition, negative attitudes and lack of knowledge towards drug misuse and abuse can aggravate physiological and mental illness which may eventually result in lifelong drug addiction. In Saudi Arabia, there was little information or a lack of studies estimating the student's knowledge, and attitudes about drug abuse. Investigating the knowledge, to seek current evidence on drug abuse and its prevention is needed to control drug dependency. Thus we aimed this study to evaluate the Saudi student’s knowledge, of drug abuse in Riyadh Saudi Arabia.

## 2 Methods and materials

### 2.1 Study design and setting

A cross-sectional study was conducted at King Saud University College of Pharmacy and Nursing in Riyadh, Saudi Arabia for a period of three months from December 2019 to February 2020. The study included entry-level male students from both Colleges, who are currently enrolled in the university. We excluded non-healthcare and senior students. The data were collected using a self-administration technique that involves paper-based, printed questionnaires. Furthermore, ethical approval was obtained by King Saud University, College of Medicine, Riyadh, Saudi Arabia. All methods were carried out according to the relevant guidelines and regulations. Informed consent was obtained from all study participants before the data collection.

### 2.2 Questionnaire design and pilot study

The anonymous questionnaires were prepared using literature from previous studies published in this regard (Arria et al., 2017, Geramian et al., 2014; J. Schulenberg et al., 2019). The questionnaire has 13 items divided into three sections (Appendix 1). The first section of the questionnaires collected demographic information from study participants, such as age, course of study, family size, parent's education (mother and father), and occupation. The second section of the survey consists of two questionnaires. The first question collected respondents' smoking habits, and the second addressed previously received information about drug abuse. These two items were graded on a yes/no scale. The third section of the study is devoted to knowledge questionnaires (drugs causing addiction, symptoms, and complications; long and short-term complications linked with drug addiction), which consist of six items with multiple choice answers. A team of professionals (Senior researcher and professor) reviewed the study questionnaires to ensure the correctness of the content, ease of use, and comprehension of the questions. A subsequent pilot research was undertaken with 30 randomly selected students. The pilot study was carried out under the supervision of a team of experts, and the results of the pilot study were not included in the main study. The Cronbach alpha value of 0.81 for knowledge surveys was suggested for use in the study.

The knowledge score for the drug-causing addition questionnaires was prepared. Each correct answer received a score of '1′, while incorrect answers received a score of '0′. The total number of items was then computed to determine overall knowledge. A similar concept was applied to other questionnaires (knowledge of short and long-term complications). The knowledge score was divided into two categories, good knowledge (students who scored more than 50 % of the total score) and poor knowledge (students who less than scored 50 % of the total score). (A. Bashatah & Wajid, 2020; A. S. Bashatah et al., 2023, Syed et al., 2022a, Syed et al., 2022b).

### 2.3 Data collection procedure

A convenience sampling strategy was used to collect data from the students. An investigator was appointed to collect data, which was collected from students during their free time after the lecture. Data were gathered until the necessary sample was obtained. Students provided written informed consent before data collection and were instructed to return completed surveys to the investigator, students who failed to do so were considered non-respondents. The Ethics Committee at King Saud University's College of Medicine in Riyadh, Saudi Arabia, approved this study.

### 2.4 Sample size estimation

**Fig. 1:**
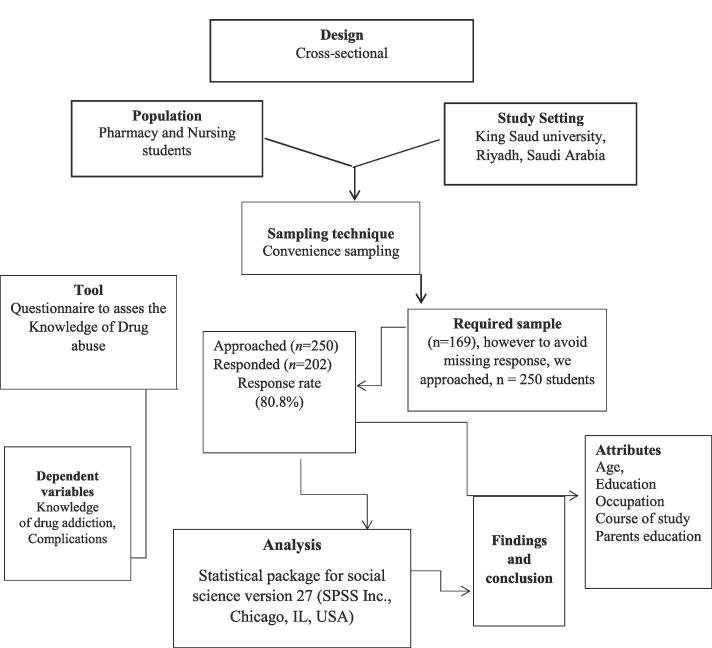
Methodological flow chart for the male pharmacy and nursing students in in Riyadh, Saudi Arabia, 2019–2020.

During the period of study, we found approximately 250 male students from both courses at the KSU campus were taking their courses, from whom we could obtain the required sample size. The sample size for this study was calculated similarly to previous studies (A. Bashatah & Wajid, 2020; A. S. Bashatah et al., 2023, Samreen et al., 2020, Syed et al., 2022c, Syed et al., 2022d, Syed et al., 2022e; Syed, Iqbal, et al., 2022; Syed et al., 2023, Syed Snr et al., 2022; Syed, Samarkandi, Sadoun, et al., 2022; Wajid et al., 2022) using an online sample size calculator with a 95 % confidence level and a pre-determined margin of error of ± 5 %. We assumed that the response distribution for each question would be 50 % because we were unsure of the potential results for each question. The calculated sample size was 169, but we decided to survey 250 male students in an attempt to ensure higher reliability (Fig. 1).

### 2.5 Statistical analysis

The data were analyzed using the statistical package for Social Science (SPSS) version 27, and a descriptive analysis such as frequency (*n*) and percentages (*%*) were calculated. The mean knowledge score was calculated for the knowledge questionnaires. The one-way ANOVA and student's *t*-test test were used, as appropriate, to assess the association between demographic characteristics. A p-value of less than 0.05 was considered statistically significant.

## 3 Results

### 3.1 Demographic characteristics

A total of 202 male students completed the questionnaires giving a response rate of (total (n = 250; 80.8 %) (Fig. 1). One hundred and nine (54 %) students were PharmD and Ninety-three (46 %) students were nursing. In this study, most of the male students from both courses were aged between 19 and 21 years. The distribution of demographic and family characteristics of the students involved in the study are presented in Table 1.

**Table 1:** Distribution of demographic and family characters of the male pharmacy and nursing students, stratified by Course of study in Riyadh, Saudi Arabia, 2019––2020 (n = 202).

Students characters	Pharmacy (n%)	Nursing (n%)	Total (n%)
Course of study	109(54)	93(46)	202(1 0 0)
Age(Years) 19–21 22–23	75(68.8) 34(31.2)	57(61.3) 36(38.7)	132(65.3) 70(34.7)
Family size* A family of 1–4 members A family of 5–9 members A family of > 10	25(22.9) 70(64.2) 10(9.2)	25(26.9) 55(59.1) 8(8.6)	50(24.8) 125(61.9) 18(8.9)
Fathers education level Illiterate Reading and writing School Education University degree	7(6.4) 6(5.5) 35(32.1) 61(56.0)	12(12.9) 4(4.3) 22(23.7) 55(59.1)	19(9.4) 10(5.0) 57(28.2) 116(57.4)
Mother's educational level* Illiterate Reading and writing School Education University degree	8(7.3) 10(9.2) 45(41.3) 46(42.2)	10(10.8) 14(15.1) 30(32.3) 38(40.9)	18(8.9) 24(11.9) 75(37.1) 84(41.6)
Father's occupation Clerk Self-employed Unemployed Retired Others	50(45.9) 9(8.3) 6(5.5) 36(33.0) 8(7.3)	49(52.7) 14(15.1) 3(3.2) 25(26.9) 2(2.2)	99(49) 23(11.4) 09(4.5) 61(30.2) 10(5.0)
Smoking status* Yes No	50(45.9) 57(52.3)	30(32.3) 63(67.7)	80(39.6) 120(59.4)
Have you received any information/course about drug abuse? * Yes No	90(82.6) 18(16.5)	61(65.6) 32(34.4)	151(74.8) 50(24.8)

*Missing response.

#### 3.1.1 Knowledge about drug causes of addiction and clinical symptoms of addicted drugs

Table 2 shows the distribution of the responses of the male students corresponding to their knowledge about drug addiction and clinical symptoms of addicted drugs. Of the entire student majority (85.3 %; 75.3 %) of the pharmacy and nursing male students thought that cocaine causes drug addiction, followed by heroin (pharmacy 80.7 %; nursing 71 %), morphine (pharmacy 75.2 %; nursing59.1 %), and opium (pharmacy 69.7 %; nursing 59.1 %). Furthermore, sleeping pills and shisha were reported by 53.2 % and 48.6 % of the pharmacy and nursing male students as shown in Table 2.

**Table 2:** Descriptive statistics of the Knowledge about drug addiction and clinical symptoms of addicted drugs among male pharmacy and nursing students in Riyadh, Saudi Arabia, 2019––2020.

Statements	Pharmacy		Nursing	
	Yes (n%)	No(n%)	Yes(n%)	No(n%)
**Drugs induce addiction** Ecstasy Sleeping pills Hashish Analgesics like ibuprofen “pills” Crack (cocaine) Antibiotics pills ShishaShires (opium extract) Morphine Heroin Cocaine Opium Psychological medication	30 (27.5)58 (53.2)74 (67.9)25 (22.9)76 (69.7)27 (24.8)53 (48.6)39 (35.8)82 (75.2)88 (80.7)93 (85.3)76 (69.7)50 (45.9)	79 (72.5)47 (43.1)35 (32.1)84 (77.1)33 (30.3)82 (75.2)56 (51.4)70 (64.2)27 (24.8)21 (19.3)16 (14.7)33 (30.3)59 (54.1)	16 (17.2)43 (46.2)51 (54.8)20 (21.5)63 (67.7)18 (19.4)33 (35.5)22 (23.7)55 (59.1)66 (71.0)70 (75.3)55 (59.1)28 (30.1)	77 (82.8)50 (53.8)42 (45.2)73 (78.5)30 (32.3)75 (80.6)60 (64.5)71 (76.3)38 (40.9)27 (29.0)23 (24.7)38 (40.9)65 (69.9)
**Complications of addictive drugs (**e.g., opium, heroin, morphine) Myosis Dry mouth Constipation Mydriasis Renal damage and failure Diarrhea Brain damage Seeing unreal images that others can’t see Hearing unreal sounds that others can’t hear	41 (37.6)47 (43.1)51 (46.8)63 (57.8)46 (42.2)26 (23.9)75 (68.8)77 (70.6)78 (71.6)	68 (62.4)62 (56.9)58 (53.2)46 (42.2)63 (57.8)83 (76.1)34 (31.2)32 (29.4)31 (28.4)	39 (41.9)36 (38.7)35 (37.6)50 (53.8)33 (35.5)32 (34.4)54 (58.1)56 (60.2)45 (48.4)	54 (58.1)57 (61.3)58 (62.4)43 (46.2)60 (64.5)61 (65.6)39 (41.9)37 (39.8)48 (51.6)

*Because of a few missing responses, the total number of responses is not the same in all categories.

Taking into consideration the signs and complications associated with stimulants, the data gathered were as follows: A total of 70.6 % of the pharmacy and, 60.2 % of the nursing male students stated that seeing unreal images that others cannot see were the main signs and complications of addicted drugs, while 71.6 % of the pharmacy and 48.4 % of the nursing male students also reported that and hearing unreal sounds that others can’t hear were the complications. The detailed responses of the students about the signs and complications of the addicted rugs are given in Table 2.

### 3.2 Association between knowledge score of drugs cause addiction and male pharmacy and nursing student’s characters in Riyadh, Saudi Arabia, 2019––2020

The mean knowledge score of drug-cause addiction was significantly higher among students aged between 19 and 20 years (t = 2.076; p = 0.039) in comparison to older students, indicating a significant difference between them(p = 0.030). Similarly, the mean knowledge score among pharmacy students was higher (7.073 ± 2.570) in comparison to nursing (5.806 ± 2.494) (t = 3.540; *p =* 0. 0001). In addition, the father’s occupation was found to be significantly associated with the mean knowledge score F = 2.667 (*p* = 0.034). The student's having previous information or a course about drug abuse and the smoking status of the students was found to have no impact on the mean knowledge score about drugs causing addiction (*t* = 1–117; *p* = 0.265; t = 0.203; *p* = 0.839) as shown in Table 3.

**Table 3:** Association between Knowledge score of drugs cause addiction and male pharmacy and nursing student’s characters in Riyadh, Saudi Arabia, 2019––2020.

Participants Characteristics	Mean	Std. Deviation (Std)	*p-*value
**Age (In years)** 19–20 21–22	6.7 5.9	2.6 2.4	*0.039*
**Course of study** Pharmacy Nursing	7.0 5.8	2.5 2.4	<0.001
**Family size** A family of 1–4 A family of 5–9 A family of > 10	6.2 6.5 7.0	2.8 2.6 2.0	0.569
**Fathers education level** Illiterate Reading and writing School Education University degree	5.4 6.4 6.7 6.5	3.2 1.8 2.5 2.5	0.265
**Mother's educational level** Illiterate Reading and writing School Education University degree	4.8 6.5 6.7 6.6	2.4 2.4 2.7 2.4	0.052
**Father's occupation** Clerk Self-employed Unemployed Retired Others	6.0 6.0 7.4 7.2 7.0	2.5 3.6 2.7 2.0 2.4	0.034
**Smoking status** Yes No	6.475 6.400	2.5 2.5	0.839
**Have you received any information or a course about drug abuse?** Yes No	6.6 6.1	2.6 2.6	0.265

Student’s *t*-test; and ANOVA.

### 3.3 Association between knowledge score of complications of addictive drugs and student characteristics

The mean knowledge score of complications of addictive drugs and student characteristics were presented in Table 4 indicating no significant association between student characters and complications of addictive drugs (p = 0.05).

**Table 4:** Association between knowledge score of complications of addictive drugs and male pharmacy and nursing student’s characters in Riyadh, Saudi Arabia, 2019––2020.

Participants Characteristics	Mean	Std. Deviation (Std)	*p-v*alue
**Age (In years)** 19–20 21–22	4.5 4.2	1.9 2.0	*0.421*
**Course of study** Pharmacy Nursing	4.7 4.1	1.8 1.9	0.707
**Family size** A family of 1–4 A family of 5–9 A family of > 10	4.7 4.4 4.0	1.9 1.9 2.1	0.410
**Fathers education level** Illiterate Reading and writing School Education University degree	4.8 4.8 4.2 4.4	2.3 1.3 1.8 1.9	0.609
**Mother's educational level** Illiterate Reading and writing School Education University degree	5.0 4.5 4.4 4.2	2.2 1.9 2.0 1.7	0.502
**Father's occupation** Clerk Self-employed Unemployed Retired Others	4.1 4.8 5.0 4.6 4.4	1.9 2.0 1.3 1.9 1.7	0.284
**Smoking status** Yes No	4.6 4.2	1.9 1.8	0.155
**Have you received any information or a course about drug abuse?** Yes No	4.4 4.3	1.8 2.2	0.197

Student’s *t*-test; and ANOVA.

### 3.4 *Students' Frequency of responses towards complications caused by stimulants (*e.g., ecstasy, shisheh)

With regards to the knowledge of complications induced by stimulants, slightly more than half of both categories of the students said that Mydriasis (Pharmacy 53.2 %; nursing 50.5 %), followed by dry mouth (Pharmacy 51.4 %; nursing 40.9 %). The distribution of the responses of the participants corresponding to their Knowledge about Complications caused by stimulants was given in Table 5.

**Table 5:** Frequency of responses towards complications caused by stimulants (ecstasy, shisheh) among male pharmacy and nursing students in Riyadh, Saudi Arabia, 2019––2020.

Statements	Pharmacy		Nursing	
	Yes (n%)	No (n%)	Yes (n%)	No (n%)
**Complications caused by stimulants** Myosis Dry mouth Mydriasis Renal damage and failure Diarrhea Brain damage Seeing unreal images that others can’t see Hearing unreal sounds that others can’t hear	47 (43.1)56 (51.4)58 (53.2)47 (43.1)29 (26.6)44 (40.4)31 (28.4)33 (30.3)	62 (56.9)53 (48.6)51 (46.8)62 (56.9)80 (73.4)65 (59.6)78 (71.6)76 (69.7)	44 (40.9)38 (40.9)47 (50.5)36 (38.7)26 (28.0)31 (33.3)28 (30.1)33 (35.5)	49 (52.7)55 (59.1)46 (49.5)57 (61.3)67 (72.0)62 (66.7)65 (69.9)60 (64.5)

### 3.5 Knowledge of students about short-term and long-term complications associated with abused drugs

The knowledge of respondents about the short-term complications of drug abuse is given in Table 6. According to findings of both groups, the students thought that euphoria and happiness were the short-term complications of drug abuse, followed by sleep disorders (pharmacy 58.7 %; nursing students 50.7 %) and aggressiveness) pharmacy 49.5 %; nursing students 51. 6 %). The detailed responses of the students are given in Table 6**.** With regards to the long-term complications of drug abuse, the large majority of both groups (74.3; 75 %%) reported that Anxiety and depression were the main complications followed by drug dependence (pharmacy 74.3 %; nursing students 64.5 %), personality disorder (pharmacy 73.4 %; nursing students 52.7 %) and aggressiveness (pharmacy 70.6 %; nursing students 69.9 %). Table 7..

**Table 6:** Descriptive statistics of the Knowledge about short-term and long-term complications of abused drugs among male Pharmacy and Nursing students in Riyadh, Saudi Arabia, 2019––2020.

Statements	Pharmacy		Nursing	
short-term complications of drug abuse	Yes (n %)	No (n%)	Yes (n%)	No (n%)
Anxiety and depression Euphoria and happiness Improved memory and learning ability Aggressiveness Raised self-confidence Pessimism Personality disorder Sleep disorder Forgetfulness Dependence to drugs	48(44.0) 70(64.2) 42(38.5) 54(49.5) 50(45.9) 31(28.4) 49(45.0) 64(58.7) 56(51.4) 50(45.9)	61(56.0) 39(35.8) 67(61.5) 55 (50.5) 59(54.1) 77(70.6) 59(54.1) 42(38.5) 53(48.6) 59(54.1)	35(37.6) 61(65.6) 25(26.9) 48(51.6) 38(40.9) 25(26.9) 39(41.9) 53(57.0) 45(48.4) 47(50.5)	58(62.4) 32(34.4) 68(73.1) 45(48.4) 55(59.1) 68(73.1) 54(58.1) 40(43.0) 48(51.6) 46(49.5)
**long-term complications of drug use**? Anxiety and depression Euphoria and happiness Improved memory and learning ability Aggressiveness Raised self-confidence Pessimism Personality disorder Sleep disorder Forgetfulness Dependence to drugs	81(74.3) 24(22.0) 25(22.9) 77(70.6) 27(24.8) 50(45.9) 80(73.4) 65(59.6) 62(56.9) 81(74.3)	28(25.7) 85(78.0) 83(76.1) 32(29.4) 82(75.2) 59(54.1) 29(26.6) 44(40.4) 46(42.2) 27(24.8)	70(75.3) 16(17.2) 25(26.9) 65(69.9) 23(24.7) 48(51.6) 49(52.7) 52(55.9) 53(57.0) 60(64.5)	23(24.7) 77(82.8) 68(73.1) 28(30.1) 69(74.2) 45(48.4) 44(47.3) 41(44.1) 40(43.0) 33(35.5)

Because of a few missing responses, the total number of responses is not the same in all categories.

**Table 7:** Association between overall knowledge score of addictive drugs and receiving education about drug abuse among male pharmacy and nursing students in Riyadh, Saudi Arabia, 2019––2020**.**

Participants Characteristics	Mean	Std. Deviation (Std)	Std. Error Mean	*t*	*p* Value
**Have you received any information or a course or training about drug abuse?** Yes No	24.2 24.2	10.5 7.2	1.4 0.6	-0.005	0.004

Student’s *t*-test.

**Fig. 2:**
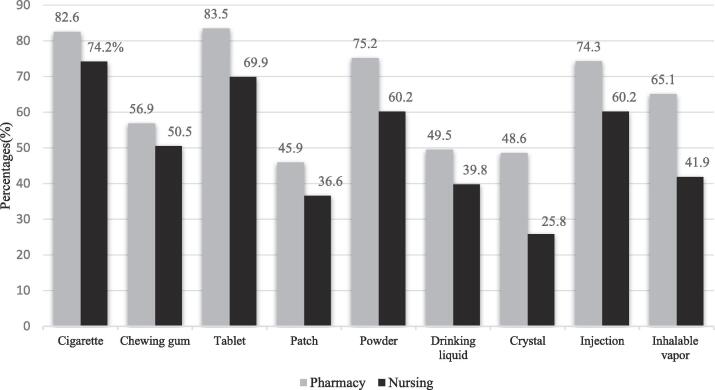
Descriptive statistics on the perceptions of male pharmacy and nursing students regarding the types of addictive substances that are readily available in Riyadh, Saudi Arabia, 2019–2020.

With regards to perceptions of students towards available forms of addictive drugs most of the students, (83.5 % of pharmacy and 76.9 % of nursing) perceived that tablet is the most common form of addictive drugs available, followed by cigarettes (82.6 % of pharmacy students and 74.2 % of nursing students) and large majorities pharmacy’s students reported that powder (75.2 %) and injections (74.3 %) are the other forms of drugs available as shown in the Fig. 2..

**Fig. 3:**
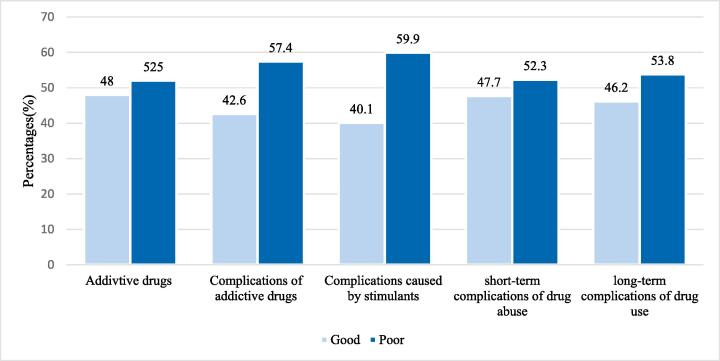
Frequency distribution of male pharmacy and nursing students' knowledge about drug-induced addictions and complications in Riyadh, Saudi Arabia, 2019 and 2020.

In this study, 52 %(n = 105) of the students reported poor knowledge of drug-induced addiction, while 48 %(n = 97) of them were found to have good knowledge. Similarly, 42.6 % of the students were found to have good knowledge of the complications of addictive drugs, and 40.1 % of them reported good knowledge of stimulants. shows the knowledge levels of drug-induced addiction among students. Detailed descriptions of the good and poor knowledge of drug addictions are given in Fig. 3**.**

**Fig. 4:**
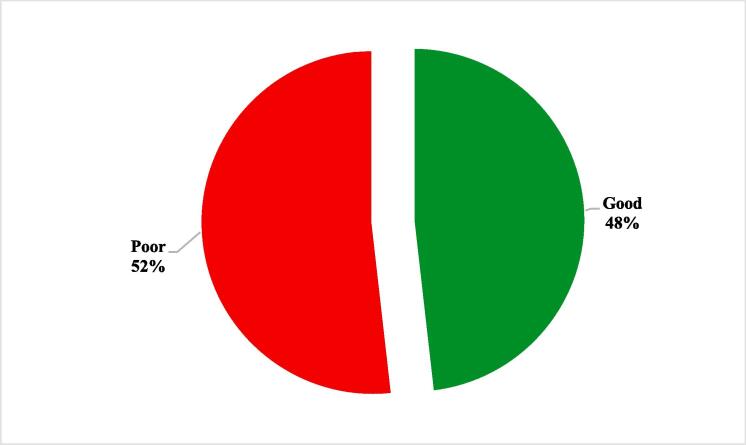
Frequency distribution of the overall knowledge levels of drug abuse among male pharmacy and nursing students in Riyadh, Saudi Arabia, 2019 and 2020.

Furthermore, 48.2 % of the students reported overall good knowledge of the concept of drug addiction, and 51.8 % of them were found to have poor knowledge of drug abuse as shown in Fig. 4.

### 3.6 Association between overall knowledge score of drug addiction and student characteristics

The mean knowledge score of drug addiction was significantly higher among students who received a drug abuse course (mean = 24.2400 ± 10.55) in comparison to those who did not (mean = 24.2329 ± 7.27), indicating a significant difference between them(p = 0.004). all other demographic variables were not significantly associated with the total knowledge score of drug addiction (p = 0.05).

## 4 Discussion

To the best of our knowledge, there have been few studies that assessed undergraduate students' knowledge of drug-causing addiction in Saudi Arabia. There was little literature found nationally and internationally about knowledge of drug-causing addiction among university students,(Arria et al., 2017; Syed, Iqbal, et al., 2022), but the majority of the literature reported on practicing pharmacists and other populations (Alenazi et al., 2023, Geramian et al., 2014, Mobrad et al., 2020, Nurmala and Muthmainnah, 2021, Siriwatanamethanon et al., 2012, Yadav and Parajuli, 2021). This study would make a significant contribution to the safety of abusers, through raising an awareness about their harmful effects among students, patients, and individuals in Saudi Arabia and other countries, and would serve as a reference for much-needed future studies. The findings could also be used by educational and healthcare institutions to develop appropriate awareness and training initiatives to improve the knowledge of pharmacists and other healthcare professionals about adverse events.

In the present study, we discovered that 54 % of pharmacy and 46 % of nursing undergraduate students participated at King Saudi University and that 48 % of the students had good knowledge regarding drug addiction. However, these findings were similar to previous findings published in Saudi Arabia and other countries(Alenazi et al., 2023, Nurmala and Muthmainnah, 2021, Siriwatanamethanon et al., 2012, Yadav and Parajuli, 2021). For instance, a prior study revealed that 57.5 % of students had poor knowledge of drug abuse and only 0.9 % had good knowledge (Yadav & Parajuli, 2021). Similarly, a study conducted in Indonesia found that students have poor knowledge and attitudes toward drug addictions(Nurmala et al., 2021). However, In Saudi Arabia, a recent study among male students revealed that the majority of the students knew the harmful effects of smoking, alcohol, and drugs(Alenazi et al., 2023). However current study results were comparable to a similar study published among practicing pharmacists to assess the knowledge, attitudes, and beliefs about drug abuse by Mobrad and his colleagues (Mobrad et al., 2020). Although knowledge of drug addiction varies from study to study and is influenced by many factors such as study method, types of respondents, and subject demographics. This could explain the difference in knowledge levels between the current study and other studies.

In this study, the mean overall knowledge score of addictive drugs among undergraduates was 6.49 + 2.607 (Median = 6; range 0–13). In addition, the mean knowledge score among pharmacy students was higher (7.073 ± 2.570) in comparison to nursing (5.806 ± 2.494) students. The difference in the knowledge score among pharmacy and nursing students in the current study might be because nursing students were less involved in dispensing medications to the patients, therefore they might not have the content about drug addiction in their curriculum at the early stages of their studies. In this current study, 82.6 % of pharmacy and 65.6 % of nursing students received some sort of information about drug abuse during their academic. These findings were similar to previous findings by Syed et al in 2022 among pharmacy and nursing undergraduates in Saudi Arabia (Syed, Iqbal, et al., 2022), where 80.4 %of the pharmacy and 67.4 % of nursing students reported having undertaken a drug misuse course in college(Syed, Iqbal, et al., 2022). However, the current study results were better than an earlier study published among Jordanian pharmacy students where approximately 64 % of the students reported a lack of information about drug abuse in their curriculum(Jaber, Bulatova, Suyagh, Yousef, & Wazaify, 2015).

Despite these evidenced reports, there were several studies underlined that pharmacists have insufficient knowledge and or skills on drug abuse. More specifically, undergraduates and employed healthcare workers are inadequately educated to organize or treat patients and collaborators with substance abuse problems(Alibrahim et al., 2012, Kenna et al., 2006). These findings necessitate critical emphasis on performing and implementing pre- and post-graduated substance abuse education and training courses and programs. Also, training or specialty in pharmacy residency programs should be delivered, and pharmacist involvement in community service and drug abuse management research should be encouraged.

This study found that 44.8 % of the pharmacy and 55.2 % of nursing students reported poor knowledge about addictive drugs. However, the students agreed that morphine, heroin, cocaine, and opium extracts cause addiction to it. Similar results were reported by Priyadarshini et al who evaluated the dentist's knowledge about drug abuse and identified that cocaine (91.6 %) heroin (75.9), cannabis (72.4), and ecstasy (36.9 %) were the most common drugs that cause addiction (Priyadarshini et al., 2019). The findings in this study differed from those of previous studies published among practicing pharmacists and dentists rather than student pharmacists and dentists(Al-Musa and Al-Montashri, 2016, Mobrad et al., 2020, Priyadarshini et al., 2019). However, the knowledge of addiction and abuse may differ from one study to another and may be influenced by several factors including the study method, types of respondents, and demographics of the subjects.

Seeing unreal images and sounds that others can't see and brain damage were the most common complications of addictive drugs identified by students. Previous reports suggested that addictive drugs potentially target the brain and affect memory, learning capability, and making judgments by changing and altering the chemical substance(WebMD, 2023). Although reports found that long-term use of addictive drugs affects the psychological and neurological conditions of the subjects(Gould, 2010, Lyvers and Yakimoff, 2003). However, there were reports by Kelley and his colleagues (Kelley, Yeager, Pepper, & Beversdorf, 2005) reported that the intake of cocaine and opioids causes insufficiencies in mental flexibility, while Moriyama and colleagues (Moriyama, Muramatsu, Kato, Mimura, & Kashima, 2006) reported that alcohol addiction affects the proper working of memory and attention. Two other studies reported that nicotine causes memory loss and declarative learning (Gould, 2010, Kenney and Gould, 2008). However, in this current study, students reported changes in psychological behaviors followed by renal damage, Mydriasis, and Myosis which are consistent with previous studies in the literature (Gould, 2010, Kelley et al., 2005, Kenney and Gould, 2008, Lyvers and Yakimoff, 2003, Moriyama et al., 2006, Priyadarshini et al., 2019).

Drug addiction was associated with serious oral and psychological health problems including decay, regressive alterations of teeth, mucosal irritation, and dryness of the mouth as reported by several studies earlier(Priyadarshini et al., 2019). In this study, one-third of the participants also reported oral health problems as a consequence of drug addiction, which is similar to an earlier study(Priyadarshini et al., 2019). Although previous reports identified that the most addictive behavior is smoking Followed by cocaine abuse, Heroin, and Alcohol while marijuana addiction occurs in about 6 % to 9 % of users(Anthony et al., 1997, Rosenberg and Anthony, 2001). Earlier Studies from different countries have reported that many drug sellers do not strictly adhere to healthcare laws and regulations when prescribing, prohibited substances although the results also revealed overprescribing in Saudi Arabia (Al-Mohamadi et al., 2013, Samreen et al., 2020; Syed, Iqbal, et al., 2022; Wajid, Al-Arifi, Al Nomay, Al Mousa, & Babelghaith, 2015). Additionally, studies revealed that the most common trend these days in both developed and other developing countries is irrational prescription, which may be the cause of the relatively high usage of substance abuse. In addition, the main causes cited for drug misuse were joy, access to drugs, and family problems, with some of them being reversible(Syed, Iqbal, et al., 2022).

There are some limitations to the current investigation. First off, the fact that the results were based on a self-administered survey may have raised the likelihood of biases such as social desirability bias or recollection bias. Second, because the data were obtained from a single Saudi Arabian university, they were not indicative of other universities and could not be applied globally. Third, because it was easier to contact male students when distributing the questionnaire, the study excluded female students and focused only on their male counterparts. Despite these drawbacks, our study proposes placing a greater emphasis on educating health college students about drug abuse and dispelling myths about using them to joy seeking to provide them with the knowledge necessary to educate the general public about drug abuse.

## 5 Conclusions

The current findings highlight that 52 % of the pharmacy and nursing students were found to have poor knowledge about drugs causing addiction. Poor knowledge was reported higher in nursing compared to pharmacy students. The knowledge score was significantly associated with age, course of study, and father's occupation. As a result, we support the implementation of educational initiatives that inform students about the negative effects of drug abuse and how to prevent their complications. Moreover, the current findings suggested the need for more efforts to increase awareness about the severe consequences of abused drugs. Furthermore, an extensive health education program on the topic in the nursing and pharmacy curriculum was warranted.

## CRediT authorship contribution statement

**Omaimah A. Qadhi:** Writing – review & editing, Writing – original draft, Software, Resources, Project administration, Methodology, Investigation, Funding acquisition, Formal analysis, Data curation, Conceptualization. **Moadeyah Mohammed Alasmari:** . **Ibrahim Nasser Alsulaihim:** . **Wajid Syed:** . **Mahmood Basil A. Al-Rawi:** .

## Declaration of competing interest

The authors declare that they have no known competing financial interests or personal relationships that could have appeared to influence the work reported in this paper.

## Data Availability

Data will be made available on request.
