# Supplementary material for: Evaluation of clinical knowledge of drugs causing addiction and associated social determinants among male pharmacy and nursing students in Riyadh, Saudi Arabia – A Cross-Sectional study
Source: Prev Med Rep. 2024 Jan 16;38:102606. doi: 10.1016/j.pmedr.2024.102606 (PMC10874849; doi:10.1016/j.pmedr.2024.102606)
Supplement: Supplementary data 1 [file mmc1.docx]

# Evaluation of Clinical Knowledge of drugs Causing Addiction and Associated Social Determinants Among Male Pharmacy and Nursing students - Across sectional questionnaires based study

**General information**

1. Age (in years): _____

2. Gender: 1. Male 2. Female

3. Family size: _____

4. Father's educational level

1. Illiterate
2. Reading and writing
3. School Education
4. University degree

5. Mother's educational level

1. Illiterate
2. Reading and writing
3. School Education
4. University degree

6. Father's occupation

1. Clerk
2. Self-employed
3. Unemployed
4. Retired
5. Others (Please write it down) ……………………………………………

7. Smoking status

1. Yes
2. No

**Knowledge related questionnaire**

1. Have you received any information about drug Abuse?

◻ Yes

◻ No

**(Each of the questions 2-7 can have more than one possible answer, so mark as many answers as you consider correct)**

**2.** Which of the following drugs causes addiction?

◻ Ecstasy

◻ Sleeping pills

◻ Hashish

◻ Analgesics like ibuprofen “pills”

◻ Crack (cocaine)

◻ Antibiotics pills

◻ Shisha

◻ Shireh (opium extract)

◻ Morphine

◻ Heroin

◻ Cocaine

◻ Opium

◻ Psychological medication (used for treatment of psychological diseases)

3. **Which of the following signs or symptoms are complications of addictive drugs (e.g., opium, heroin, morphine…)?**

◻ Myosis

◻ Dry mouth

◻ Constipation

◻ Mydriasis

◻ Renal damage and failure

◻ Diarrhea

◻ Brain damage

◻ Seeing unreal images that others can’t see

◻ Hearing unreal sounds that others can’t hear

**4. Which one of the following complications is caused by stimulants (e.g., ecstasy, shisheh)?**

◻ Myosis

◻ Dry mouth

◻ Mydriasis

◻ Renal damage and failure

◻ Diarrhea

◻ Brain damage

◻ Seeing unreal images that others can’t see

◻ Hearing unreal sounds that others can’t hear

5. **Which of the following is the short-term complication of drug abuse?**

◻ Anxiety and depression

◻ Euphoria and happiness

◻ Improved memory and learning ability

◻ Aggressiveness

◻ Raised self-confidence

◻ Pessimism

◻ Personality disorder ا

◻ Sleep disorder

◻ Forgetfulness

◻ Dependence to drugs

6. **Which of the following are the long-term complications of drug use**?

◻ Anxiety and depression

◻ Euphoria and happiness

◻ Improved memory and learning ability

◻ Aggressiveness

◻ Raised self-confidence

◻ Pessimism

◻ Personality disorder

◻ Sleep disorder

◻ Forgetfulness

◻ Dependence to drugs

7.**What form of drugs is available?** **(you can choose more than one answer)**

◻ Cigarette

◻ Chewing gum

◻ Tablet

◻ Patch

◻ Powder (sugar or salt)

◻ Drinking liquid

◻ Transparent crystal

◻ Injection

◻ Inhalable vapor of some liquids
